# In Silico Design and Validation of OvMANE1, a Chimeric Antigen for Human Onchocerciasis Diagnosis

**DOI:** 10.3390/pathogens9060495

**Published:** 2020-06-22

**Authors:** Cabirou Mounchili Shintouo, Robert Adamu Shey, Derrick Neba Nebangwa, Kevin K. Esoh, Nkemngo Francis Nongley, Joel Ebai Nguve, Philippe Giron, Léon Mutesa, Luc Vanhamme, Jacob Souopgui, Stephen Mbigha Ghogomu, Rose Njemini

**Affiliations:** 1 Department of Gerontology, Faculty of Medicine and Pharmacy, Vrije Universiteit Brussel, Laarbeeklaan 103, B-1090 Brussels, Belgium; Cabirou.Mounchili.Shintouo@vub.be; 2 Frailty in Ageing Research Group, Vrije Universiteit Brussel, Laarbeeklaan 103, B-1090 Brussels, Belgium; 3 Department of Biochemistry and Molecular Biology, Faculty of Science, University of Buea, P.O. Box 63 Buea, Cameroon; robeshey@ulb.ac.be (R.A.S.); neba.nebangwa@ubuea.cm (D.N.N.); joelebai@gmail.com (J.E.N.); 4 Department of Molecular Biology, Institute of Biology and Molecular Medicine, IBMM, Université Libre de Bruxelles, Gosselies Campus, 126040 Gosselies, Belgium; Luc.Vanhamme@ulb.be; 5 Division of Human Genetics, Department of Pathology, University of Cape Town, Health Sciences Campus, Anzio Rd, Observatory 7925, South Africa; ESHKEV001@myuct.ac.za; 6 Department of Microbiology and Parasitology, Faculty of Science, University of Buea, P.O. Box 63 Buea, Cameroon; nkemngo.francis@gmail.com; 7 Department of Parasitology and Medical Entomology, Centre for Research in Infectious Diseases (CRID), Yaounde, Cameroon; 8 Laboratory of Medical and Molecular Oncology, Oncology Research Center, Faculty of Medicine and Pharmacy, Vrije Universiteit Brussel, Laarbeeklaan 103, B-1090 Brussels, Belgium; Philippe.Giron@vub.be; 9 Centre for Human Genetics, College of Medicine and Health Sciences, University of Rwanda, KG11 Ave, Kigali 4285, Rwanda; l.mutesa@ur.ac.rw

**Keywords:** *Onchocerca volvulus*, OvMANE1, chimeric antigen, IgG, diagnosis

## Abstract

The public health goal of onchocerciasis in Africa has advanced from control to elimination. In this light, accurate diagnosis is necessary to determine treatment endpoints and confirm elimination, as well as to conduct surveillance for the identification of any possible recrudescence of the disease. Currently, the monitoring of onchocerciasis elimination relies on the Ov-16 test. However, this test is unable to discriminate between past and active infections. Furthermore, about 15–25% of infected persons are reported to be negative for the Ov-16 test, giving a misleading sense of security to false-negative individuals who might continue to serve as reservoirs for infections. Therefore, we opted to design and validate a more sensitive and specific chimeric antigen (OvMANE1) for onchocerciasis diagnosis, using previously reported immunodominant peptides of *O. volvulus,* the parasite responsible for the disease. In silico analysis of OvMANE1 predicted it to be more antigenic than its individual peptides. We observed that OvMANE1 reacts specifically and differentially with sera from *O. volvulus* infected and non-infected individuals, as well as with sera from communities of different levels of endemicity. Moreover, we found that total IgG, unlike IgG4 subclass, positively responded to OvMANE1, strongly suggesting its complementarity to the Ov-16 diagnostic tool, which detects Ov-16 IgG4 antibodies. Overall, OvMANE1 exhibited the potential to be utilized in the development of specific diagnostic tools—based on both antibody capture and antigen capture reactions—which are indispensable to monitor the progress of onchocerciasis elimination programs.

## 1. Introduction

Human onchocerciasis—or “river blindness”—is one of the most devastating yet neglected tropical diseases caused by a filaria nematode (*Onchocerca volvulus*) and transmitted by the frequent bites of infective black flies of the genus *Simulium* [1]. In 2017, approximately 20.9 million people were infected with *O. volvulus* worldwide; 1.15 million had vision loss and about 14.6 million had a skin disease, with more than 99% living in Africa [2]. The disease contributes to higher rates of epilepsy, lower life expectancy and consequently higher mortality and great economic loss in the affected populations, making it a significant public health concern as well as a serious cause of social stigmatism [3,4]. 

Success registered by the African Program for Onchocerciasis Control (APOC) to reduce morbidity and transmission rates in endemic areas has led to a shift in the health goal of onchocerciasis in Africa from control to elimination [5,6]. The Expanded Special Project for Elimination of Neglected Tropical Diseases (ESPEN) is functioning towards reaching the onchocerciasis elimination milestone in designated African countries by 2020 and in a minimum of 80% of African countries by 2025 [7]. However, moving from control to elimination is a tedious process, as programs must shift from identifying communities with symptomatic individuals to pin-pointing communities with infected but asymptomatic individuals [8]. Thus, diagnostic tests such as palpation of nodules and skin-biopsy for microfilaria and approaches like Rapid Epidemiological Mapping of Onchocerciasis which were vital for identifying areas of prime importance for prevention of onchocerciasis activities, may not be adequate tools for elimination programs [9,10,11].

The current monitoring of the disease elimination efforts relies on the absence of *O. volvulus* DNA in pools of *Simulium* black flies and the absence of IgG4 antibodies to Ov-16 antigen as approved by the World Health Organization [12]. However, the Ov-16 serological test—though reported to have excellent specificity—has moderate sensitivity (80%) for microfilaria positive individuals and is unable to discriminate between past and active infections [13,14]. Moreover, about 15–25% of infected persons are reported to be negative, due partly to genetic restrictions [13,15]. This suggests that the Ov-16 test systemically produces a significant number of false-negative individuals who might continue to serve as reservoirs for infections, ensuring the continuous transmission of the disease following certification of elimination [13]. Also, to meet the concerns of single-antigen tests such as the Ov-16 test, multi antigenic tests using synthetic peptide mixture have been exploited [16]. However, the use of a synthetic peptide cocktail often results in the competitive binding of the different peptides and poor binding of some of them is often observed in solid-phase, which affects the sensitivity and specificity of the test [17]. Hence, there is a die need to engineer molecular tools of antigen capture diagnosis for the fight against human onchocerciasis.

Chimeric antigens have been posited to constitute a potential tool for more sensitive and specific serodiagnosis of diseases [17,18]. Indeed, the use of chimeric antigens for immunodiagnosis has been reported for many infectious diseases including helminth infection, toxoplasmosis, HIV-1, Chagas disease and malaria with increased sensitivity and specificity [17,19,20,21,22,23,24,25]. On the other hand, the proteome-wide linear epitope repertoire from microarray studies with onchocerciasis patients have led to the recognition of immunodominant antigenic peptides that could be further harnessed in the development of a diagnostic test [26]. Therefore—in our global working strategy aiming at generating reliable antibodies for the development of antigen capture tests to diagnose onchocerciasis—we opted in the first step to design and validate a recombinant chimeric antigen, OvMANE1, using previously reported immunodominant peptides [26] of *O. volvulus*. 

## 2. Results

In silico analysis of OvMANE1 chimeric antigen predicted it to be more antigenic than its individual peptides. We observed that OvMANE1 chimeric antigen reacts specifically and differentially with sera from *O. volvulus* infected and non-infected individuals, as well as with sera from communities of different levels of endemicity. Moreover, we found that total IgG, unlike IgG4 subclass, positively responded to OvMANE1 chimeric antigen, strongly suggesting its complementarity to the Ov-16 diagnostic tool, which detects Ov-16 IgG4 antibodies. Overall, OvMANE1 chimeric antigen exhibited convincing characteristics supporting its potential use in both the development of antibody capture test and generation of reliable antibodies required for antigen capture diagnostic tool for onchocerciasis.

### 2.1. Peptide Selection and Stage-Specific Expression

**Figure 1:**
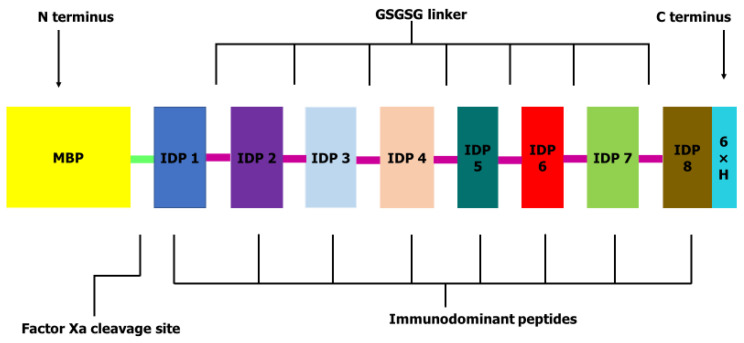
Schematic presentation of OvMANE1 chimeric antigen: The 153 amino acid long antigen sequence consisting of selected immunodominant peptides (IDP) fused using GSGSG linkers (purple lines). As a result of the expression in a pMAL vector, OvMANE1 chimeric antigen is flanked with the maltose binding protein (MBP) and a 6x-His-tag was coupled to the C-terminus for downstream characterization.

Eight peptide sequences that had previously revealed high performance in immunomic assays with immunodominant motifs, ^1^PxxTQE^6^ and ^1^DGxDK^5^, sensitivities of 80.0–95.2% and specificities of 92.2–100.0% were retrieved from previously reported data [26] and used to design the OvMANE1 chimeric antigen (see Table S1). WormBase gene expression data indicate that all the selected peptides were expressed in all the parasite stages. The eight selected immunodominant peptides (IDP) were fused to a flexible GSGSG linker which maximized epitope recognition of a fusion protein [27] to obtain the OvMANE1 chimeric antigen construct. A 6xHis tag was coupled to the C-terminus to aid in purification and identification (153 amino acids). As a result of the employed pMAL expression vector, OvMANE1 chimeric antigen was flanked at its N-terminus by the maltose binding protein (MBP) with a factor Xa cleavage site. The final construct obtained was 560 amino acids in length (see Figure 1).

### 2.2. Antigenicity, Physicochemical Properties and Solubility of OvMANE1 Chimeric Antigen

Focusing on OvMANE1 protein without the flanking MBP tag protein, the antigenicity prediction of the final construct by ANTIGENpro is 0.917040 and by VaxiJen 2.0 server is 0.8524 using a parasite model at a threshold of 0.5. The results indicate that the generated construct is antigenic in nature. Furthermore, the OvMANE1 chimeric antigen was predicted to be more antigenic than its individual peptides that were used for the construct except for OVOC3954 antigen which showed slightly higher antigenicity than the chimera based on ANTIGENpro server prediction. Notwithstanding, the VaxiJen 2.0 server predicted the chimera to be more antigenic than the OVOC3954 antigen. Also, the antigenicity of OvMANE1 was similar to that of OvMANE1_MBP on the ANTIGENpro serve but higher on the VaxiJen 2.0 server (see Table 1).

Various physicochemical properties of OvMANE1 chimeric antigen with and without the MBP tag were predicted by the Expasy ProtParam online server. The molecular weights of OvMANE1 and OvMANE1_MBP chimeric antigens were predicted to be 15.8 kDa and 60.4 kDa, respectively. The theoretical isoelectric point (pI) values were 4.81 and 4.96 for OvMANE1 and OvMANE1_MBP, respectively. Based on these pI values, the protein was predicted to be acidic in nature with or without the MBP tag. The instability indexes (II) were predicted to be 31.13 and 24.39 for OvMANE1 and OvMANE1_MBP, respectively, classifying the protein as stable with or without the MBP tag. The estimated aliphatic indexes were predicted to be 48.43 for OvMANE1 and 70.79 for OvMANE1_MBP indicating thermostability since higher values of the aliphatic indices are related to protein thermostability [28]. Thus, MBP was predicted to increase the thermostability of OvMANE1 chimeric antigen. The predicted Grand average of hydropathicity was −0.888 for OvMANE1 and −0.543 for OvMANE1_MBP which indicates that the protein with or without the MBP tag is hydrophilic in nature and can interact with water molecules [29]. Protein-Sol server predicted OvMANE1 and OvMANE1_MBP proteins to be soluble upon expression with a score of 0.890 and 0.604 respectively.

### 2.3. Secondary and Tertiary Structures

**Figure 2:**
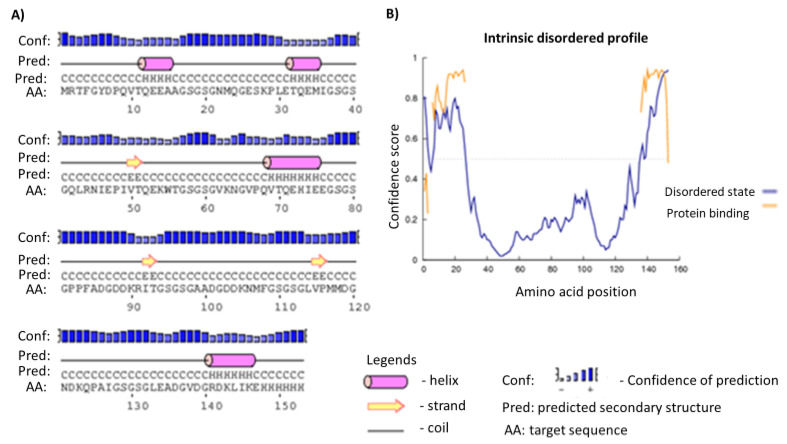
PSI-blast based secondary structure PREDiction (PSIPRED) graphical representation of secondary structural features of OvMANE1 chimeric antigen construct showing (**A**) alpha-helix (12.0%), beta strands (2.0%) and coils (84.0%) as well as (**B**) Table 100.0% of positions are predicted as disordered).

**Figure 3:**
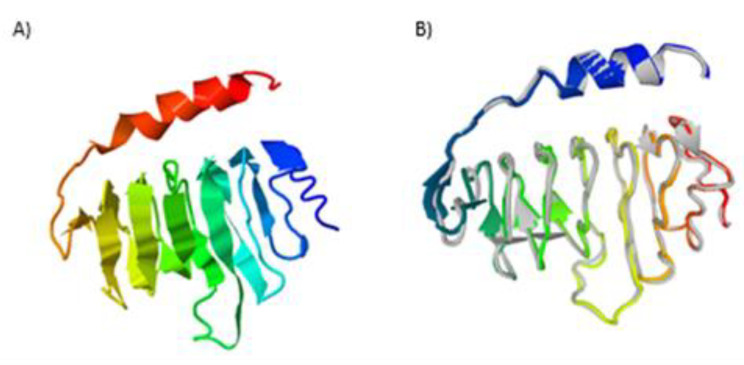

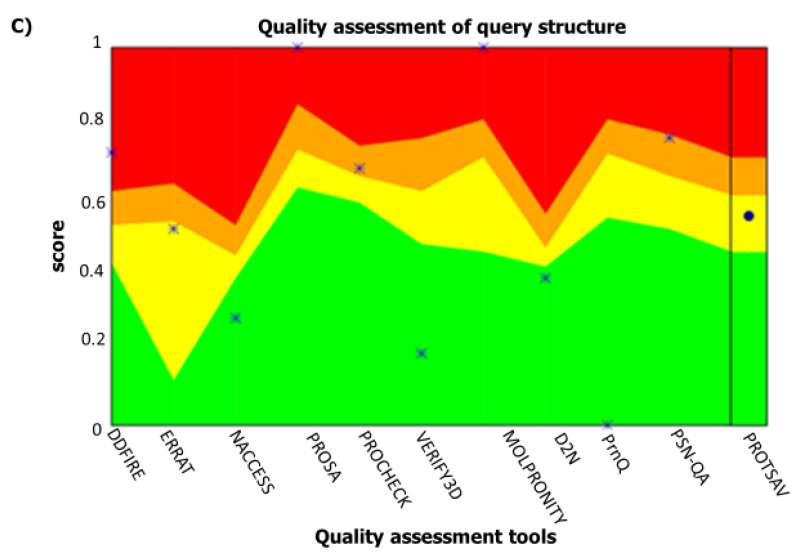
Protein modelling, refinement and validation. (**A**) The final 3D model of OvMANE1 chimeric antigen gotten after homology modelling on I-TASSER. (**B**) Refined 3D structure overlay (colored) on the ‘crude model’ (gray) by GalaxyRefine. (**C**) Refined model validation using ProTSAV predicted the refined structure to be within the range of 2–5 Å estimated root mean squared deviation (RMSD).

The final construct without the MBP tag was predicted to contain 12% alpha-helix, 2% beta-strand and 84% coil (see Figure 2A). Moreover, concerning solvent accessibility, 4% of the residues were exposed, 84% in the medium while 9% were predicted to be buried. A total of 153 amino acid residues (100%) were predicted as disordered by the RaptorX Property server (see Figure 2B). Five three-dimensional (3D) models of the chimeric antigen were predicted by I-TASSER based on 10 threading templates, top amongst which have protein data bank identifications; 6emkG, 3jbmA, 1dd3A, 5aftV and 5n8pA. Seven of the ten chosen templates showed good alignment as qualified by their *Z-score* values. The five predicted models had *C-score* values ranging from −4.29 to −3.12. The model with the lowest *C*-score from the homology modelling was selected for further refinement (Figure 3A). This model had an estimated TM-score of 0.36 ± 0.12 with an estimated root mean squared deviation (RMSD) of 11.9 ± 4.4 Å. Subsequent refinement of the initial antigen model on the ModRefiner server followed by GalaxyRefine server yielded five models. Based on model quality scores for all refined models, the second model was found to be the best based on different parameters including GDT-HA (0.9477), RMSD (0.421) and MolProbity (2.454) (see Figure 3B). The quality and potential errors in a crude 3D model were verified by ProTSAV. The final protein model was selected with the RMSD in the range 2–5 Å (see Figure 3C).

### 2.4. Mass Expression and Purification of Recombinant OvMANE1 Chimeric Antigen from Bacterial Cells

**Figure 4:**
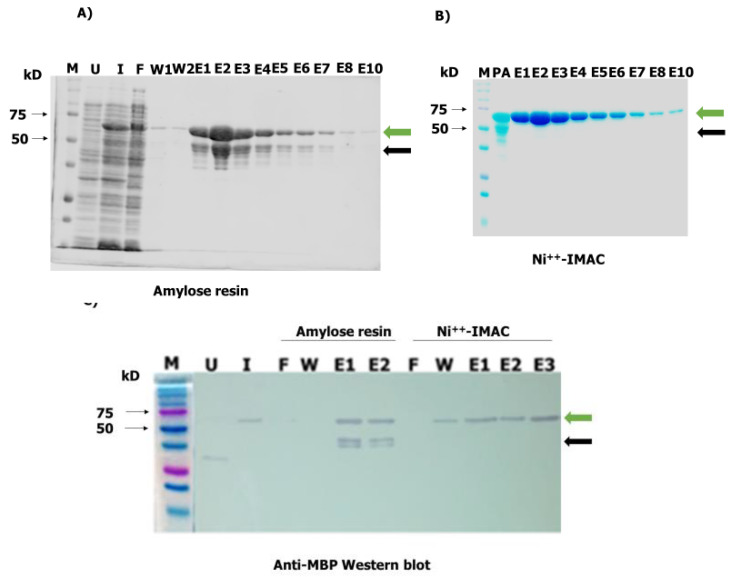
Expression and purification of OvMANE1 chimeric antigen. OvMANE1_MBP initially purified on amylose resin (**A**) and further purified using Ni++-IMAC column (**B**), was resolved on a 12.5% Sodium Dodecyl Sulfate–Polyacrylamide Gel Electrophoresis (SDS-PAGE) gel and stained with Coomassie blue. (**C**) Anti-MBP monoclonal antibody detected the protein on nitrocellulose membranes at a molecular weight of approximately 60.4 kDa corresponding to the expected size of OvMANE1_MBP chimeric antigen. Green and black arrows indicate the positions of OvMANE1_MBP chimeric antigen and degradation products respectively. M = protein ladder, U = uninduced, F = flow-through, W = wash, E = Eluate, PA = purified from amylose resin.

OvMANE1 chimeric gene was synthesized and cloned into pMAL-c5X expression vector by GenScript (Leiden, The Netherlands). The recombinant plasmid was used to transform NEB^®^ Express Competent *E. coli* cells (New England Biolabs, Ipswich, MA, USA). Expression as a maltose binding protein (MBP) fusion protein (OvMANE1_MBP, 60.4 kDa) was subsequently induced in transformed bacterial cultures. The full recombinant chimeric protein (green arrow, Figure 4A) was purified using amylose resin (New England Biolabs, Ipswich, MA, USA). In order to eliminate degradation products (black arrow), an additional purification step was performed using the Ni^++^-immobilized-metal affinity chromatographic (IMAC) (see Figure 4B). The molecular weight and purity of OvMANE1_MBP (60.4 kDa of which 44.6 kDa is contributed by the MBP tag and factor Xa cleavage site) were assessed by Sodium Dodecyl Sulfate–Polyacrylamide Gel Electrophoresis (SDS-PAGE) and Western blot using anti-MBP monoclonal antibodies (see Figure 4C).

### 2.5. The Humoral Response to OvMANE1 Chimeric Antigen Divulges Its Diagnostic Potentials

**Figure 5:**
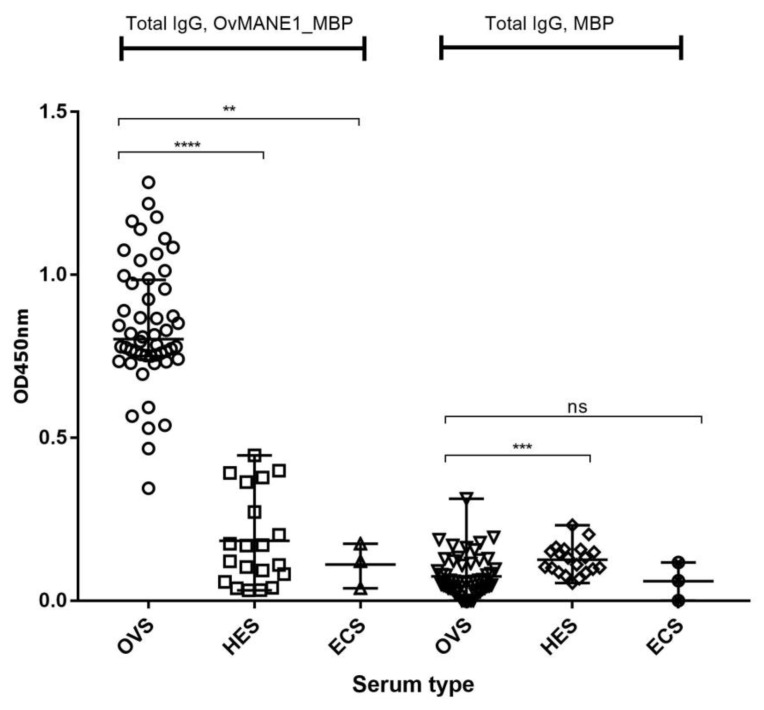
Humoral immune response to OvMANE1 chimeric antigen by enzyme-linked immunosorbent assay (ELISA). Purified OvMANE1_MBP chimeric antigen or MBP (control) was used to coat microtiter plates. The microtiter plates were blocked and incubated with serum from either OVS, HES or ECS, followed by incubation with goat anti-human IgG peroxidase conjugate. The microtiter plates were revealed using TMB and the optical density (OD) was read at 450 nm. The OD values were plotted against the different serum types. OVS = *O*. *volvulus* serum (*n* = 52), HES = Hypo-endemic serum (*n* = 20), ECS = European control serum (*n* = 03). A Kruskal-Wallis test was used to compare the groups.

In order to validate OvMANE1 chimeric antigen as a putative diagnostic tool, the humoral immune response against its constitutive epitopes was assessed in patients. Total IgG responses were screened by indirect ELISA in sera from infected and non-infected individuals. A discriminatory immune response to the constituents of OvMANE1 chimeric antigen was observed between serum samples from infected and non-infected individuals. The mean optical density (OD) read at 450 nm for *O*. *volvulus* infected sera (OVS) was significantly different from that of hypo-endemic sera (HES) from Rwanda—which is reported as a low-risk zone for *O. volvulus* infection [30]—and European control sera (ECS), with *p* < 0.0001 and *p* = 0.0049 for HES and ECS, respectively (see Figure 5). The area under the receiver operating curve (AUC) was found to be very high, with a value of 0.9952 and a *p* < 0.0001 (see Table 2) indicating both high sensitivity and specificity. As expected, total IgG responses to the purified MBP used as negative control were very low and could not discriminate between sera from the infected and uninfected persons.

### 2.6. Analysis of Total IgG Responses to OvMANE1 Chimeric Antigen in Communities of Different Levels of Endemicity

**Figure 6:**
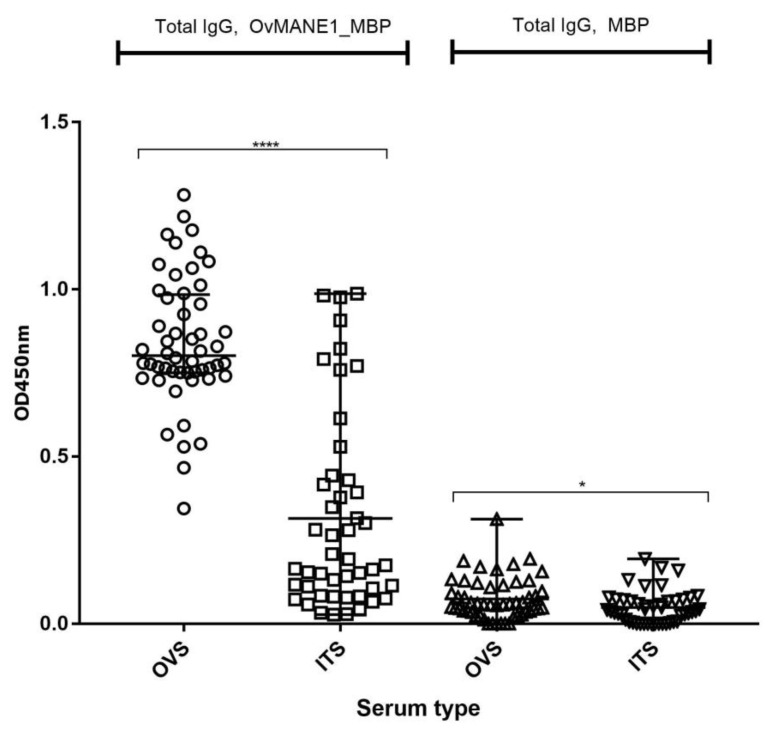
Analysis of IgG responses to OvMANE1 chimeric antigen in communities of different levels of endemicity. Purified OvMANE1_MBP chimeric antigen or MBP (control) was used to coat microtiter plates. Microtiter plates were blocked and later incubated with serum from *O*. *volvulus* serum (OVS) or Ivermectin treated serum (ITS) followed by incubation with goat anti-human IgG peroxidase conjugate. The microtiter plates were revealed using TMB, the optical density (OD) read at 450 nm and OD values were plotted against the different serum types. OVS, *n* = 52, ITS, *n* = 47. Mann-Whitney U test was used to compare the responses.

In order to assess if OvMANE1 chimeric antigen can be used as a possible biomarker for monitoring the success of elimination programs, we investigated by ELISA the correlation between the humoral immune response to OvMANE1 chimeric antigen in different onchocerciasis endemic communities. As shown in Figure 6, OvMANE1 chimeric antigen significantly discriminated between OVS from the hyperendemic region of Kombone and ivermectin treated persons (ITS) of the onchocerciasis-near elimination region of Bandjoun. As expected, responses to the MBP tag protein were low and could not discriminate between these two communities. These results suggest that anti-OvMANE1 chimeric antigen immune response could be employed in distinguishing communities of different levels of endemicity.

### 2.7. Cross-Reactivity Test with Related Nematode

**Figure 7:**
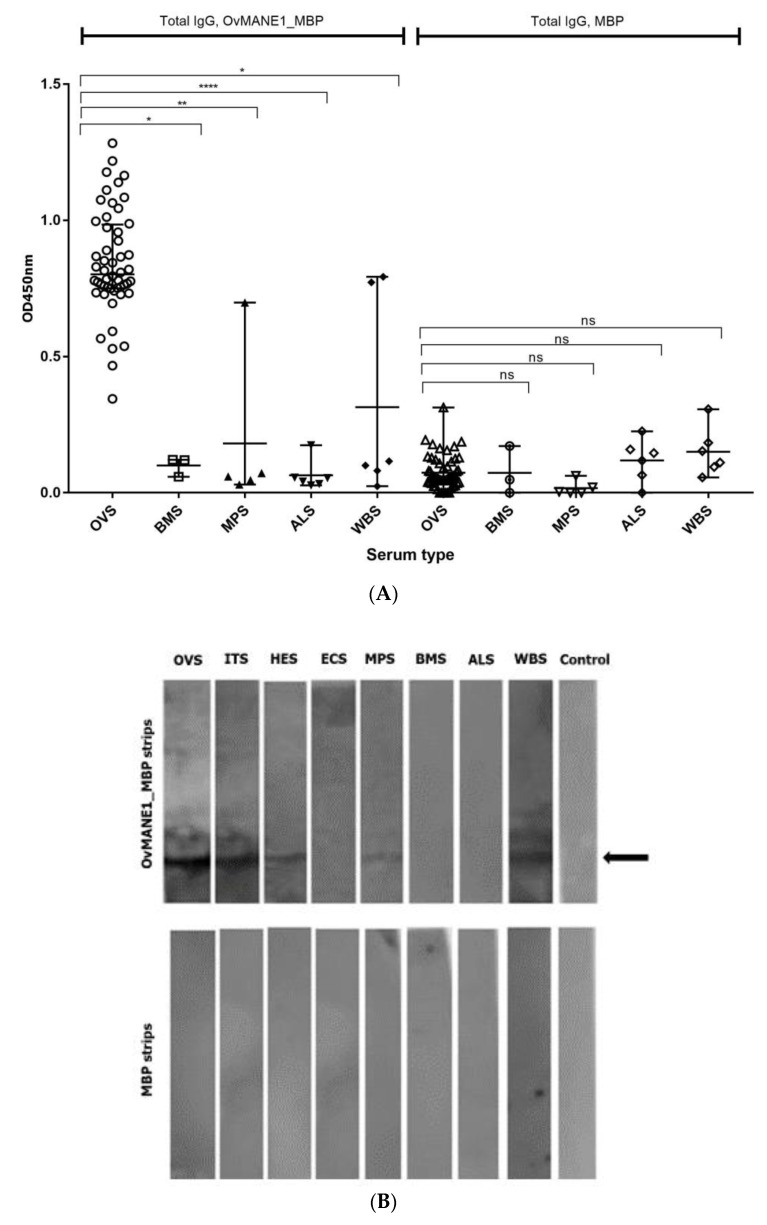
Analysis of the humoral immune responses of related nematode sera to OvMANE1 chimeric antigen. (**A**) Purified OvMANE1_MBP chimeric antigen or MBP (control) was used to coat microtiter plates. Microtiter plates were blocked and later incubated with serum from OVS, BMS, MPS, ALS or WBS followed by incubation with goat anti-human IgG peroxidase conjugate. The microtiter plates were revealed using TMB, the optical density (OD) was read at 450 nm and OD values were plotted against the different serum types. OVS = *O*. *volvulus* serum (*n* = 52), BMS = *B*. *malayi* serum (*n* = 03), MPS = *M*. *perstans* serum (*n* = 06), ALS = *A*. *lumbricoides* serum (*n* = 06) and WBS = *W. bancrofti* serum (*n* = 06). (**B**) OvMANE1_MBP antigen recognition patterns. Western blotting experiments were performed to address the reaction patterns of OvMANE1_MBP with pools of serum samples form *O. volvulus* patients and related nematodes samples, using the MBP-tag as a control. Serum pools were made as follows: OVS = *O. volvulus* serum (*n* = pool of 10 serum samples), ITS = Ivermectin treated serum (*n* = pool of 10 serum samples), HES = Hypo-endemic serum (*n* = pool of 10 serum samples), ECS = European control serum (*n* = pool of 3 serum samples), MPS = *M. perstans* serum (*n* = pool of 6 serum samples), BMS = *B. malayi* serum (*n* = pool of 3 serum samples), ALS = *A. lumbricoides* serum (*n* = pool of 6 serum samples) and WBS = *W. bancrofti* serum (*n* = pool of 6 serum samples). The black arrow indicates the position of OvMANE1 chimeric protein on the strip.

Possibility of cross-reaction with OvMANE1 chimeric antigen in individuals infected with other related nematodes was investigated using serum samples from patients infected with *Brugia malayi*, *Mansonella perstans*, *Ascaris lumbricoides and Wuchereria bancrofti* by ELISA. In contrast to our previous observed cross-reaction of this set of serum samples with Ov28CRP/OvGM2AP, an excretory secretory product of *O. volvulus* [31] OvMANE1 chimeric antigen significantly discriminated onchocerciasis sera from that of related nematodes (see Figure 7A). 

In order to investigate if IgG responses to OvMANE1_MBP correlated with antigen recognition profile, Western blotting experiments were carried out using specific nematodes serum samples. As shown in Figure 7B, we observed one single band on OvMANE1_MBP strip with *O. volvulus* serum (OVS) pool, ivermectin treated serum (ITS), hypo-endemic serum (HES), *M. perstans* serum (MPS) and *W. bancrofti* serum (WBS) but no band was observed with European control serum (ECS), *B. malayi* serum (BMS) and *A. lumbricoides* serum (ALS). No band was observed for the MBP strip.

### 2.8. Total IgG but Not the IgG4 Subclass Responded Positively to OvMANE1 Chimeric Antigen

**Figure 8:**
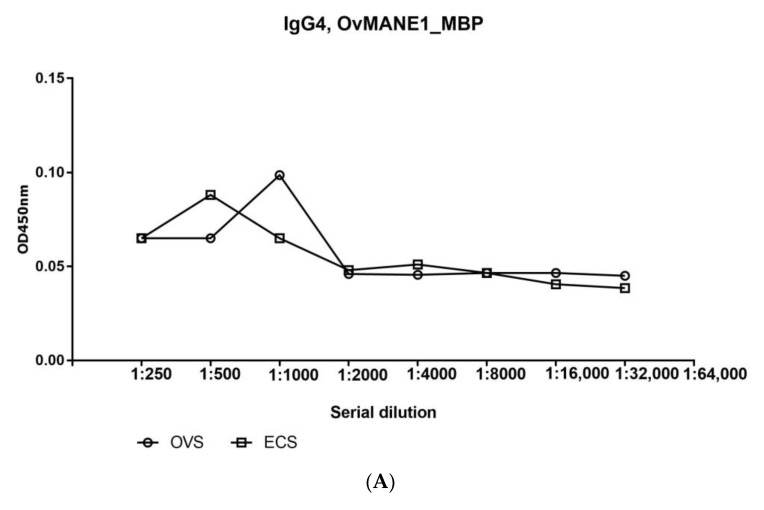

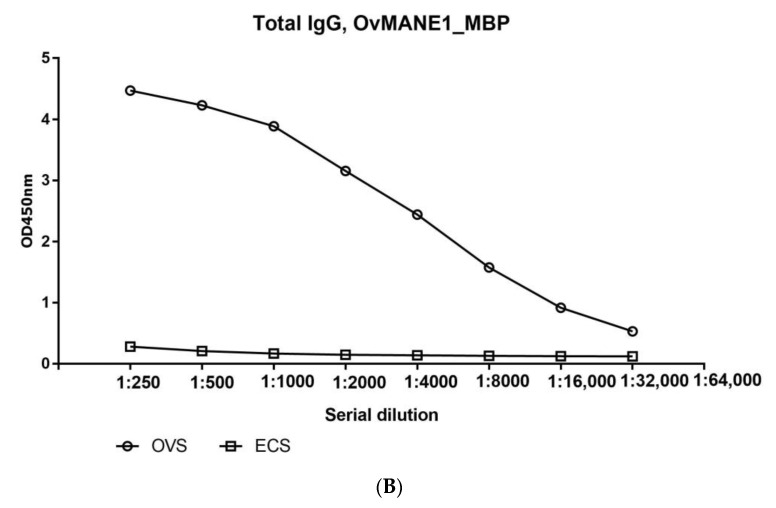
Analysis of humoral immune response to OvMANE1 chimeric antigen using sera pools from infected and non-infected individuals. Purified OvMANE1_MBP chimeric antigen was used to coat microtiter plates. Microtiter plates were blocked and incubated with serum pools from either OVS or ECS at a dilution from 1:250 to 1:32,000 followed by incubation with (**A**) mouse monoclonal anti-Human IgG4 (HRP) or (**B**) goat anti-human IgG peroxidase conjugate as the secondary antibody. The microtiter plates were revealed using TMB, the optical density (OD) was read at 450 nm and OD values were plotted against the different serum types. OVS = *O. volvulus* serum (*n* = pool of 10 infected serum samples), ECS = European control serum (*n* = pool of 3 control serum samples).

For onchocerciasis, measurements of IgG isotypes in filarial infections has revealed that IgG4 accounts for up to 95% of the IgG response to these infections [32] rendering IgG4 a marker of onchocerciasis infection. Thus, the IgG4 humoral immune response to OvMANE1 chimeric antigen was evaluated by ELISA using serum pools from infected and non-infected individuals. We observed that OvMANE1_MBP chimeric antigen failed to react with IgG4 subclass as testified by the serial dilution curves (see Figure 8A) as well as to discriminate between sera pools from infected and non-infected individuals. In contrast, when total IgG was tested in the same serially diluted serum samples, OvMANE1 chimeric antigen, as expected, reacted strongly with serum pool from *O. volvulus* infected individuals and could discriminate the serum pools from infected and non-infected individuals (see Figure 8B).

## 3. Discussion

For ESPEN to achieve its goal of elimination of onchocerciasis in at least 80% of African countries by 2025, there is a need for accurate diagnosis for complete disease elimination mapping, routine monitoring and evaluation of mass drug administration programs [33]. Currently, the monitoring of elimination efforts for onchocerciasis relies on the entomological evaluation of the parasite in the vector using the O-150 PCR poolscreen technique and serological evaluation of the parasite in children below 10 years using the Ov-16 test [12]. However, the Ov-16 test is based on antibody capture and cannot discriminate past from active onchocerciasis infection. Moreover, it has inadequate sensitivity to allow its optimal use in low prevalence settings [8] and reports indicate that a test composed of a single antibody for epidemiological surveillance purposes is not fully sufficient to determine true infection prevalence [26,34]. Furthermore, 15–25% of infected persons are reported to be negative due to genetic restrictions [15], suggesting that the Ov-16 test produces false-negative individuals who might continue to transmit the disease [13].

To circumvent these shortcomings, OvMANE1 chimeric antigen was designed and validated as a potential biomarker for the development of a diagnostic tool for human onchocerciasis. The designed chimeric antigen was predicted to be antigenic using the ANTIGENpro and Vaxijen v2.0 servers. Overall, this chimeric antigen proved to be a better antigen than each of the individual peptides used in the chimeric construct. Notwithstanding, the superior antigenicity of OvMANE1 chimeric antigen needs to be validated biochemically via Western blot and/or ELISA. Also, the predicted characteristics of OvMANE1 chimeric antigen make it a good candidate for use in the production of antibodies against target antigens of *O. volvulus*, which could be applied in an antigen-capture test to discriminate past from ongoing *O. volvulus* infections. These predicted properties of the chimeric antigen are consistent with the profile observed during the expression and purification of the antigen.

Secondary structure analyses of OvMANE1 chimeric antigen revealed that the protein consists predominantly of coils (84%), with 100% of its residues being disordered. Natively unfolded protein regions and alpha-helical coiled-coils peptides are of significant importance for the design of new peptide-based diagnostic tests. Indeed, synthetic peptides with these two structural forms have the ability to fold into their native structure and be identified by antibodies naturally induced by infectious agents [35]. On the other hand, disordered proteins fulfill essential biological functions. For example, the increased plasticity of disordered proteins favor their binding to numerous and structurally distinct targets [36]. Moreover, the 3D structure model of the chimeric antigen improved profoundly after refinement. Nevertheless, this structure needs to be validated via crystallization of the chimeric antigen.

The patients’ humoral immune responses to the constituents of the designed OvMANE1 chimeric antigen were validated using the recombinant protein expressed in bacterial cells, OvMANE1_MBP. Analysis of the total IgG response to OvMANE1 chimeric antigen revealed its ability to differentiate between infected and non-infected individuals. The specificity of a test is of high importance [37]: thus, a cutoff value of 0.456 was defined for OvMANE1 chimeric antigen that corresponds to 100% specificity and 98% sensitivity. With these settings, the chimeric antigen discriminated between ongoing infections and treated cases. This finding is beneficial in the framework of the evaluation of onchocerciasis elimination programs. Also, one of the essential components for an excellent diagnostic antigen is its ability to uniquely detect a target parasite species, a very challenging daunting task owing to the high sequence homology amongst genes from related nematodes [38]. OvMANE1 chimeric antigen did not cross-react with sera of closely related nematodes. Therefore, OvMANE1 chimeric antigen may have a potential application in the specific diagnosis of human onchocerciasis based on our findings herein. Responses to the MBP tag that was linked to OvMANE1 chimeric antigen were quite low for all the tested samples and could not discriminate between sera from infected and uninfected persons. This result suggests that the humoral immune responses detected to the constituents of recombinant OvMANE1_MBP antigen were not contributed to by the MBP tag.

The antigen recognition pattern of OvMANE1_MBP revealed by Western blot correlates with the observed humoral immune responses to the constituents of OvMANE1_MBP chimeric antigen. Thus, the ELISA signals were from OvMANE1_MBP reactions and not of any contaminant. The singled bands observed in OVS, ITS and HES were expected since these serum samples come from onchocerciasis hyper or hypo endemic zones. Single bands were also found with MPS and WBS consistent with the ELISA result—which showed some high responders in ELISA experiments—and suggesting that these serum samples might have been obtained from patients co-infected with *O. volvulus*. No band was observed for ECS, BMS and ALS suggesting no cross reactivity with OvMANE1_MBP chimeric antigen. Finally, it was observed, as expected that strips coated with MBP-tag as control revealed no signal with all the different serum pools tested, strongly suggesting that signals obtained were specific to OvMANE1 antigen. Overall, OvMANE1 chimeric antigen appears suitable for further characterization in terms of specificity as a serodiagnostic candidate for human onchocerciasis.

Reports indicate that the humoral immune response against parasitic antigens is frequently dominated by IgG4 responses [37,39]. Thus, IgG4 responses to the constituents of OvMANE1 chimeric antigen were evaluated using serum pools from infected and non-infected individuals. The humoral immune response of IgG4 to OvMANE1 chimeric antigen was weak and could not discriminate between pooled sera from infected and non-infected individuals. This is consistent with the results obtained when the individual peptides were used, as the IgG4 levels in response to the different individual peptides were low or absent in all tested individuals [26]. A possible explanation why there is no IgG4 response to the chimeric antigen might be due to the difference between conformational epitopes on intact surface antigens. This hypothesis is in line with reports from other studies on Ov-20 immunodominant antigen, where only the intact protein could be recognized by IgG4 antibodies, while IgG1, IgE and IgM antibodies were shown to also bind smaller fragments of the antigen [40]. On the other hand, IgG1 and IgG3 were reported to be the dominant isotypes of the individual peptides used to construct OvMANE1 chimeric antigen [26]. Thus, IgG1 and IgG3 humoral immune responses to OvMANE1 chimeric antigen need to be evaluated. Nevertheless, total IgG immune response to the constituents of recombinant OvMANE1 chimeric antigen could discriminate between the serum pools of infected and non-infected individuals.

The findings of the present study should be interpreted within its limitations. First, we did not validate biochemically that OvMANE1 chimeric antigen is more antigenic than its component peptides. Secondly, we did not provide any data regarding the seroreactivity against individual peptides and OvMANE1 as well as binding of different isotypes like IgG and IgM, which is important in determining the early/late infection condition. Thirdly, given the small sample size in some of the subgroups, it is possible that our study was not sufficiently powered to guarantee the superior specificity of OvMANE1 chimeric antigen.

In conclusion, OvMANE1 chimeric antigen is immunogenic with higher sensitivity and specificity as compared to its individual constituent peptides. The chimeric antigen may therefore be developed for use as a field-deployable diagnostic test for onchocerciasis and for monitoring the success of onchocerciasis elimination programs.

## 4. Materials and Methods

### 4.1. Ethical Considerations

The study was done in adherence to the set of principles of the Helsinki Declaration and the protocols adopted were reviewed and approved by the Cameroon Bioethics Initiative (CAMBIN) Ethics Review and Consultancy Committee (ERCC) (N˚CBI/443/ERCC/CAMBIN). Administrative authorization was sought from the Cameroon Ministry of Public Health (N˚631–1315). Informed consent forms were provided and well explained to all participants who took part in the study. All the participants willingly signed the consent form prior to participation in the study. Participation was entirely voluntary and individuals were free to withdraw at their discretion. Participants’ confidentiality was respected during data collection, analysis and reporting.

### 4.2. Serum Samples

Trained medical personnel examined all the participants involved in the study and blood samples were obtained from patients residing in the endemic region of Kombone Health Area within the Mbonge Health district in the South West Region of Cameroon. These onchocerciasis patients (OVS, *n* = 52) were chosen on the basis of a confirmed presence of clinical manifestation of onchocerciasis and/or presence of microfilaria in skin biopsies. Blood samples were also collected from an onchocerciasis-near elimination region—ivermectin treatment serum (ITS, *n* = 47)—in the Bandjoun Health District in the West Region of Cameroon. In the West Region of Cameroon, treatment of the disease has been ongoing for more than fifteen years [41]. Kamga, et al. [42] evaluate the parasitological status of individuals in Bandjoun in 2017 and characterized the infection status of the individuals. We collected blood samples from patients who were microfilaria negative and did not have any clinical manifestation of onchocerciasis. Blood samples obtained from individuals residing in an onchocerciasis hypo-endemic region (HES, *n* = 20) of Huye, Rwanda—considered a low-risk zone for onchocerciasis [30]—and from European subjects (ECS, *n* = 3), were used as controls. The blood samples were processed to obtain serum by employing an established protocol [43], diluted 1:2 in glycerol and stored at −20 °C. Serum samples from individuals infected with other nematode infections such as *Brugia malayi* (BMS, *n* = 3), *Mansonella perstans* (MPS, *n* = 5), *Ascaris lumbricoides* (ALS, *n* = 6) and *Wuchereria bancrofti* (WBS, *n* = 6), were obtained from the filarial repository, thanks to the laboratory of Dr. Steven Williams.

### 4.3. Peptide Selection, Stage-Specific Expression and Construction of OvMANE1 Chimeric Antigen

Peptides were selected based on previous studies carried out on the *O. volvulus* peptide repertoire [26]. Protein sequences for the selected peptides were assessed on WormBase (https://www.wormbase.org) for stage-specific expression. A flexible linker (GSGSG) was used as a spacer between epitope sequences [44]. A 6xHis tag was coupled to the C-terminus of the chimeric antigen to get the final antigen construct. Consistent with the employed pMAL expression vector, the chimeric construct was flanked at its N-terminus by MBP with a factor Xa cleavage site.

### 4.4. Prediction of Antigenicity, Physicochemical Properties and Solubility of OvMANE1 Chimeric Antigen

ANTIGENpro and VaxiJen v2.0 servers were used to predict the antigenicity of OvMANE1 chimeric antigen. ANTIGENpro (http://scratch.proteomics.ics.uci.edu/) is a sequence-based, alignment-free and pathogen-independent predictor of protein antigenicity. It is the first predictor of the whole protein antigenicity trained using reactivity data obtained by protein microarray analysis [45]. VaxiJen v. 2.0 server uses a new alignment-independent method, according to an auto cross-covariance (ACC) transformation of protein sequences into uniform vectors of principal amino acid properties. The accuracy of VaxiJen v. 2.0 varies from 70% to 89%, depending upon the organism targeted [45]. Different physicochemical properties of OvMANE1 chimeric antigen were determined using the Expasy ProtParam online server (http://web.expasy.org/protparam/) [46]. It computes various physicochemical properties such as amino acid composition, theoretical pI, instability index, aliphatic index, molecular weight and grand average of hydropathicity. The solubility of the chimeric antigen was evaluated using the Protein-Sol server (https://protein-sol.manchester.ac.uk/). The server uses available data for *Escherichia coli* protein solubility in a cell-free expression system, to predict the solubility of a protein [47].

### 4.5. Prediction of Secondary and Tertiary Structures

The secondary structure of the chimeric antigen was predicted by the PSIPRED server (http://bioinf.cs.ucl.ac.uk/index.php?id=779). PSIPRED uses a simple and accurate secondary structure prediction method, incorporating two feed-forward neural networks that perform an analysis on output obtained from PSI-BLAST and it determines the percentage of helix, stands and coils [46]. The RaptorX Property web server (http://raptorx.uchicago.edu/StructurePropertyPred/predict/) was later used to predict properties of the secondary structure of OvMANE1 chimeric antigen. The server employs the deep convolutional neural fields to predict secondary structure, solvent accessibility and disorder regions simultaneously [48].

Homology modelling of OvMANE1 chimeric antigen was performed using the Iterative Threading ASSEmbly Refinement (I-TASSER) server (https://zhanglab.ccmb.med.umich.edu/I-TASSER/). I-TASSER generates 3D atomic models from multiple threading alignments and iterative structural assembly simulations starting from an amino acid sequence [1]. A two-step refinement of the 3D model obtained for OvMANE1 chimeric antigen was done on ModRefiner (https://zhanglab.ccmb.med.umich.edu/ModRefiner/) followed by the GalaxyRefine server (http://galaxy.seoklab.org/cgi-bin/submit.cgi?type=REFINE). The ModRefiner server does construction and refinement of protein structures from Cα traces based on a two-step, atomic-level energy minimization, resulting in overall improvements in both global and local structures with more accurate side-chain positions, fewer atomic overlaps and better hydrogen-bonding networks [1]. Refinement using the GalaxyRefine server was achieved by subsequent overall relaxation and repeated structural perturbation by molecular dynamics simulation [49]. The refined model was validated using ProTSAV server.

### 4.6. Codon Optimization, Cloning, Expression and Purification of OvMANE1 Chimeric Antigen

To express OvMANE1 chimeric antigen in *E. coli* (strain K12), the Java Codon Adaptation Tool (JCat) server (http://www.jcat.de) was used to reverse translate and optimize the codon since codon usage of *E. coli* differs from the native host—*O. volvulus*—where the sequence of OvMANE1 chimeric antigen is derived. The codon optimized gene sequence of OvMANE1 chimera was synthesized and cloned into pMAL-c5X vector by GenScript (Leiden, The Netherlands).

The recombinant OvMANE1_PMAL-c5X plasmid was used to transform NEB^®^ Express Competent *E. coli* cells (New England Biolabs, Ipswich, MA, USA). Expression was induced using 0.3 mM Isopropyl β-d-1-thiogalactopyranoside at 37 °C for 2 h with shaking at 200 rpm and the protein was expressed as a fusion with MBP. The bacterial cells expressing protein were pelleted from the culture medium, resuspended in column buffer (per liter: 20 mL 1.0 M Tris-HCl, pH 7.4, 11.7 g NaCl, 2.0 mL 0.5 M EDTA and 0.7 mL β-mercaptoethanol) and frozen overnight at −20 °C. Thereafter, the sample was placed in an ice-water bath and lysed by sonication in short pulses of 10 s at 40% amplitude for 3 min. Centrifugation at 20,000× *g* for 20 min was then performed and the supernatant diluted to 1:6 with column buffer. This was followed by protein purification using the amylose resin (New England Biolabs, Ipswich, MA, USA), according to the standard protocol. Due to the presence of degraded proteins, a second round of purification for pooled amylose resin purified fractions was achieved by using Ni++-IMAC columns (GE Healthcare, Diegem, Belgium) following standard protocol. The purified protein was resolved on SDS-PAGE and its identity confirmed by Western blot using anti-MBP monoclonal antibodies (New England Biolabs, Ipswich, MA, USA). The concentrations of purified proteins were determined using the Bio-Rad protein assay kit (Bio-Rad, Carlsbad, CA, USA).

For Western blots, 2 μg of protein samples were run on a 12% Tris-glycine polyacrylamide gel (Bio-Rad, Carlsbad, CA, USA) and subsequently transferred to Hybond-C Extra nitrocellulose membranes (GE Healthcare, Diegem, Belgium). Blocking was done using 5% non-fat dry milk in TBS-NP40 overnight at 4 °C followed by incubation with anti-MBP monoclonal antibodies (1:5000) for 1 h 30 min. After three changes of wash buffer (TBS + 0.005% NP40) at 5 min intervals each, membranes were incubated with ALP-conjugated secondary antibodies (1:5000) for 1 h 30 min and detected with nitro blue tetrazolium/5-bromo-4-chloro-3-indolyl phosphate (Sigma, St. Louis, MI, USA). All antibody incubation steps were done at room temperature. The chimeric antigen recognition pattern was evaluated as described above except for pooled sera from onchocerciasis patients, ivermectin treated patients, people from hypo-endemic regions, European control participants, *M. perstans* patients, *B. malayi* patients, *A. lumbricoides* patients or *W. bancrofti* patients that were used as primary antibody.

### 4.7. Serological Characterization of OvMANE1 Chimeric Antigen

IgG response to OvMANE1 chimeric antigen was investigated by indirect ELISA using infected and non-infected sera. Optimal antigen/antibody concentrations were determined by the checkerboard titration method. Maxisorp 96 well microtiter plates (Nunc, Roskilde, Denmark) were coated with 2 μg/mL of purified OvMANE1_MBP chimeric antigen overnight at 4 °C. Plates were washed three times with wash buffer (PBS + 0.05% Tween 20) and blocked with TBS supplemented with 3% Bovine Serum Albumin (BSA) (Sigma, St. Louis, MI, USA) for 1 h 30 min at room temperature. The plates were then washed and incubated with the various serum samples as the primary antibody at a dilution of 1:2000 for 2 h at room temperature. Thereafter, plates were washed and incubated with goat anti-human IgG (Fc Specific) peroxidase conjugate (Sigma, St. Louis, MI, USA) as the secondary antibody at a dilution of 1:5000 for 1 h 30 min at room temperature. After a final wash, the chromogenic substrate 3,3’,5,5’-tetramethylbenzidine (TMB, Sigma, St. Louis, MI, USA) was added for 10 min at room temperature. The reactions were stopped with 3 M hydrochloric acid after which the OD was read at 450 nm using the iMark microplate reader (BIORAD, Irvine, CA, USA). All antibody dilutions were done in a blocking buffer (TBS supplemented with 3% BSA).

IgG4 responses were determined using serum pools from ten infected patients or three European control. ELISA was performed as described above with the exception of incubating the pooled sera as primary antibodies at a dilution from 1:250 to 1:32,000 and using mouse monoclonal anti-human IgG4 Fc (HRP) antibody (Abcam, Cambridge, UK) as the secondary antibody.

### 4.8. Data Analyses 

The normality of distributions was assessed using a Shapiro-Wilk test. Comparisons of two groups were done using Mann-Whitney U test and for three or more groups using Kruskal-Wallis test. The discriminatory performance of total IgG was assessed using receiver operating curve analyses. The area under the receiver operating curve (AUCs) were evaluated using the trapezoid method. Standard errors of AUCs were calculated as previously described [50]. Exact 95% confidence intervals for the AUCs were determined using a binomial approach. An optimal cutoff value was selected according to the highest Youden’s index and the sensitivities, specificities with 95% confidence intervals were then calculated for the selected cutoff value. Scatter plots were generated using Graph Pad Prism 7.0 (La Jolla, CA, USA) and data were expressed as median with interquartile range. A *p*-value < 0.05 was considered statistically significant.

## Figures and Tables

**Table 1:** Antigenicity of OvMANE1 chimeric antigen compared to its individual peptides, as assessed and scored according to the two indicated servers.

Peptide	Antigenicity	
	ANTIGENpro	VaxiJen 2.0
OvMANE1	0.917040	0.8524
OvMANE1_MBP	0.909324	0.5816
OVOC5897	0.899261	0.5319
OVOC4989	0.798654	0.5678
OVOC5528	0.759612	0.5324
OVOC9141	0.596235	0.4489
OVOC7266	0.898052	0.6243
OVOC1743	0.243291	0.5040
OVOC1920	0.764529	0.5875
OVOC3954	0.953580	0.5964

**Table 2:** Receiver operating curve (ROC) values for IgG responses to OvMANE1 chimeric antigen and diagnostic accuracy parameter.

	Total IgG	
**ROC Curve Analysis**	ROC curve area (AUC)	0.9952
	95% CI of AUC	0.9845 to 1.006
	*p*-value (against AUC = 0.5)	<0.0001
**Diagnostic Accuracy Parameter**	Cut off value	0.456
	Sensitivity (%) (95% CI)	98.08 (89.74% to 99.95%)
	Specificity (%) (95% CI)	100 (83.16% to 100%)

## Supplementary Materials

The following are available online at https://www.mdpi.com/2076-0817/9/6/495/s1, Table S1: Selected peptide sequences and their corresponding sensitivity and specificity.
