# Supplementary material for: In Silico Design and Validation of OvMANE1, a Chimeric Antigen for Human Onchocerciasis Diagnosis
Source: Pathogens. 2020 Jun 22;9(6):495. doi: 10.3390/pathogens9060495 (PMC7350323; doi:10.3390/pathogens9060495)
Supplement: Supplementary file 1 [file pathogens-09-00495-s001.pdf]

pathogens

Supplementary Materials: The following are available online at www.mdpi.com/xxx/s1, Table S1: Selected peptide sequences and their corresponding sensitivity and specificity.

Table S1: Selected peptide sequences and their corresponding sensitivity and specificity.

Protein ID	Sequence	Sensitivity (%)	Specificity (%)
OVOC5897 (IDP 1)	RTFGYDPQVTQEEAA	95.5	96.3
OVOC4989 (IDP 2)	NMQGESKPLETQEMI	95.2	94.3
OVOC5528 (IDP 3)	QLRNIEPIVTQEKWT	95.2	95.3
OVOC9141 (IDP 4)	VKNGVPQVTQEHIEE	95.2	92.7
OVOC7266 (IDP 5)	PPFADGDDKRIT	80.0	97.4
OVOC1743 (IDP 6)	AADGDDKNMF	80.0	100
OVOC1920 (IDP 7)	LVPMMDGNDKQPAI	86.7	92.2
OVOC3954 (IDP 8)	LEADGVDGRDKLIKE	80.0	98.4

The sensitivity and specificity of the selected peptides were obtained from data previously reported by Lagatie et al. [1].
